# Nomogram for Predicting the Relationship between the Extent of Visceral Pleural Invasion and Survival in Non-Small-Cell Lung Cancer

**DOI:** 10.1155/2021/8816860

**Published:** 2021-05-24

**Authors:** Fan Wang, Pei Li, Fengsen Li

**Affiliations:** ^1^The Fourth Affiliated Hospital of Xinjiang Medical University, Urumqi 833000, Xinjiang, China; ^2^The Second Affiliated Hospital of Xinjiang Medical University, Urumqi 833000, Xinjiang, China; ^3^Kelamayi City Dushanzi People's Hospital, 833600, Xinjiang, China

## Abstract

**Objective:**

Although visceral pleural invasion (VPI) has already been incorporated into the TNM staging system, few studies have been conducted to evaluate the prognostic value of the extent of VPI for the survival of non-small-cell lung cancer (NSCLC) patients. Thus, we utilized the Surveillance, Epidemiology, and End Results (SEER) database to assess the correlation between the extent of VPI and survival in NSCLC.

**Methods:**

We identified and incorporated the extent of VPI to build a prognostic nomogram in this study. Patients in the SEER database diagnosed with NSCLC (*n* = 87,045) from 2010 to 2015 were further analyzed and randomly assigned into either the training group (*n* = 60,933) or validation group (*n* = 26,112). Clinical variables were calculated by means of multivariate Cox regressions and incorporated into the predictive model. Subsequently, the accuracy and discrimination of nomogram were further assessed through the concordance index (C-index), calibration curves, and Kaplan–Meier curves.

**Results:**

Multivariate analysis demonstrated that the extent of visceral pleural invasion was an independent and unfavorable prognostic factor. The C-indexes of the training and validation groups were 0.772 (95% CI: 0.770–0.774) and 0.769 (95% CI: 0.765–0.773), respectively, which revealed that the nomogram had sufficient credibility and stable predictive accuracy. The calibration curve displayed consistency between the actual and predictive values in both training and validation groups.

**Conclusion:**

The prognostic nomogram with the extent of VPI could offer an accurate risk evaluation for patients with NSCLC. Independent external validation of this research should be conducted in the future.

## 1. Introduction

Lung cancer is a group of clinically and histologically heterogeneous diseases and is the leading cause of cancer-related mortality worldwide [1]. Lung cancer is diagnosed in approximately 2.1 million people annually worldwide, with about 1.8 million people die of this disease [2]. Among patients, approximately 85% of patients were classified as having non-small-cell lung cancer (NSCLC). The 5-year survival rate for NSCLC patients ranges from 4% to 17% due to stage and geographic differences [3].

Visceral pleural invasion (VPI) has been identified as a poor prognostic factor in NSCLC and was first adopted as a *T* descriptor in the 5th edition of the TNM classification criteria in the 1970s [4]. According to the International Association for the Study of Lung Cancer (IASLC) standard classification, PL0 has been defined as there is no evidence of visceral pleural invasion surpassing the elastic layer; PL1 has been identified as the invasion surpassing the elastic layer; PL2 represents the invasion to the pleural surface; and PL3 indicates the invasion to the parietal pleural, thoracic wall, or both [5, 6]. In the recently updated 7th and 8th editions of the American Joint Committee on Cancer (AJCC) staging system, VPI regarded as a non-size-based T2 factor, which was previously determined to be T1 stage, is now considered to be T2a stage [6, 7]. Although the prognostic value of VPI has been widely recognized, some studies have argued that the extent of VPI has a limited impact on the survival of lung cancer patients [8, 9]. It remains controversial whether the extent of VPI affects the survival of NSCLC patients.

We must assess clinicopathological prognosis factors, including histological classification, tumor differentiation, tumor size, and so on, to estimate the accurate prognosis and deliver personalized treatment strategies for NSCLC patients. How to determine the subdivision level of VPI has some limitations in clinical application, which is usually due to being diagnosed by imaging features and clinical judgment rather than pathological diagnosis. VPI in NSCLC patients is usually determined by the tumor site, radiographic findings, and other clinical characteristics; therefore, patients have variable clinical outcomes. Moreover, although VPI has been incorporated into the existing TNM staging system of AJCC, the relationship between the subdivision of VPI and prognosis in the NSCLC patients continues to have a great deal of ambiguity. Based on these reasons, we are proposing the use of additional prognostic models to evaluate the survival outcomes of these patients.

Nomography, as a prognostic tool, can integrate predictive factors to establish a statistical model and has been extensively applied to predict the survival outcomes of various cancer patients [10, 11]. So far, no relevant nomogram involving the subdivisions of VPI has been established to predict prognosis in NSCLC patients. Therefore, we aimed to establish a nomogram that can visualize the survival prediction results of NSCLC patients with different extents of VPI by analyzing data from the Surveillance, Epidemiology, and End Results (SEER) database.

## 2. Methods

### 2.1. Data Abstraction

The SEER database, which has primary sources of population-based cancer statistics in the USA, capturing approximately 97% of cancer incidence and covering about 28% of the USA population in 17 SEER registries, is maintained by the National Cancer Institute (NCI) [12] (https://seer.cancergov/). We extracted the data of non-small-cell lung cancer patients from the SEER database using the SEER^*∗*^Stat program (v 8.2.1). The classification of VPI was based on the proposal of the International Association for the Study of Lung Cancer (IASLC) [5]. According to the SEER data term, CS Site-Specific Factor 2, the subdivision level of VPI confirmed by pathology was introduced in this database in 2010. Therefore, we retrieved only the data of lung cancer patients diagnosed by pathology from 2010 to 2015 and initially obtained a total of 443,960 patients. The included criteria to further screen the patients were as follows: (1) all included subjects needed to be histopathologically confirmed as NSCLC; (2) histological subtypes, including squamous cell carcinoma, adenocarcinoma, large-cell carcinoma, and bronchioalveolar carcinoma, were assigned according to the World Health Organization (WHO) classification system; (3) definite diagnosis of NSCLC was primary and unique; (4) the degree of visceral pleural invasion and survival status was definite; (5) comprehensive clinical information, such as race, sex, age, marital status, *T* stage, *N* stage, *M* stage, tumor grade, survival month, vital status, and the extent of VPI, were recorded. The excluded criteria were as follows: (1) incomplete clinical data involving survival time; *T*, *N*, and *M* stage; and the subdivision level of VPI and (2) histologically confirmed as small-cell lung cancer. The eligible data of NSCLC patients were randomly divided into either the training group (*n* = 60,933) or the validation group (*n* = 26,112) at a ratio of 7:3.

### 2.2. Variable Definition

The study variables were recorded as follows: gender (female and male), race (black, white, and others), *T* stage (*T*0, *T*1, *T*2, *T*3, and *T*4), N stage (*N*0, *N*1, *N*2, and *N*3), *M* stage (*M*0 and *M*1), tumor grade (well-differentiated, Grade I; moderately differentiated, Grade II; poorly differentiated, Grade III; and undifferentiated, Grade IV), histologic classification (adenocarcinoma, squamous cell carcinoma, large-cell carcinoma, and bronchioalveolar carcinoma), marital status (married (including common law), divorced, single (never married), widowed, and unknown), the extent of VPI (no evidence of visceral pleural invasion based on clinical and/or pathological judgment, PLx; tumor confined within the lung parenchyma or does not fully invade beyond the elastic layer based on histopathological evidence, PL0; tumor penetrates beyond the elastic layer based on histopathological evidence, PL1; tumor penetrates to the surface of the visceral pleura based on histopathological evidence, PL2; and tumor extends to the parietal pleura based on histopathological evidence, PL3). The primary end point was overall survival (OS), which was calculated starting from the date of diagnosis to death by any cause.

We obtained permission to retrieve the SEER research data files with the reference number 16828-Nov2017. The research data used in this study did not involve the subjects or individual identification information. Therefore, this study did not require informed consent and ethical approval.

### 2.3. Statistical Analysis

We used R software version 3.5.1 to perform statistical analysis and generate graphics (R Foundation for Statistical Computing, Vienna, Austria). The continuous variables were converted to categorical variables and calculated using the Chi-squared test. The survival curve for OS was displayed using the Kaplan–Meier method, and differences were tested using a log-rank test stratified based on the prognostic factors. The Cox proportional hazards multivariate regression was used to analyze further the variables with *P*-values less than or equal to 0.05, which had been calculated using the univariate analysis.

The graphical nomogram was obtained using the logistic regression model from the training group using the R package rms. The maximum points for each variable were set to 100. The discrimination and predictive powers of the nomogram model were evaluated using the concordance index (C-index) [13]. The discrimination ability of the prognosis model gradually increased with the increase in scores, the value of the C-index with 0.5 representing a random chance and 1.0 representing a fully corrected discrimination ability. The calibration plots of the nomogram for 3- and 5-year OS, which used bootstraps of 200 resamples, were constructed to evaluate the consistency between the predictive and actual survivals. A two-tailed *P*-value of less than 0.05 was considered statistically significant.

## 3. Results

### 3.1. Characteristics of Patients

**Figure 1:**
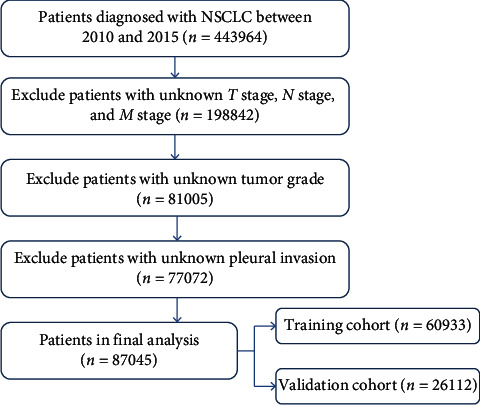
The flowchart of patients included and grouped.

This study initially included a total of 87,045 lung cancer patients from the SEER research database from 2010 to 2015 that subsequently was randomly divided into either the training group (*n* = 60,933) or the validation group (*n* = 26,112) at a ratio of 7:3. The selection process is presented in a detailed flow chart (Figure 1), and the demographic characteristics are listed in Table 1. Among these patients, 42,199 (48.5%) were female, and 44,846 (51.5%) were male. The majority of the initially included patients were married elderly patients (>60 years) and had adenocarcinoma and a tumor grade of III.

### 3.2. Prognostic Factors Associated with OS

The variables of the training group including gender, age, race, marital status, histology classification, tumor grade, TNM stage, and the subdivision of the VPI, were incorporated into the univariate analysis. All selected variables with a *P*-value of less than 0.05 using the univariate analysis were determined as risk factors and were subsequently further analyzed using Cox proportional hazards multivariate regression. Ultimately, the analysis results showed that these variables, obtained by the univariate and multivariate analysis, were independent prognostic factors (Table 2).

### 3.3. Nomogram Construction and Validation

**Figure 2:**
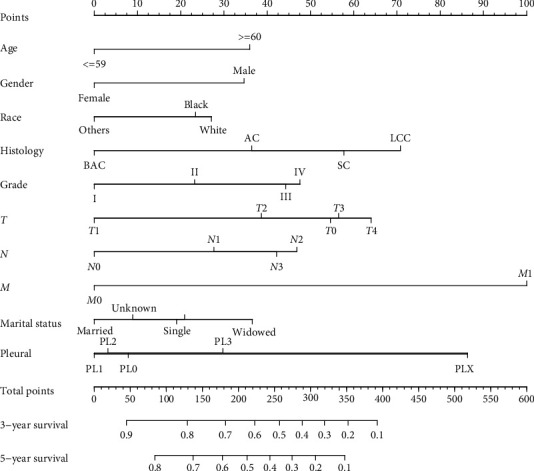
Nomogram for survival predicted probability. Each variable was individually evaluated for every patient and given a score subsequently. The higher scores obtained by summing the points of each variable from different patients indicate the poorer survival probability. Abbreviations: BAC, bronchioalveolar carcinoma; AC, adenocarcinoma; LCC, large-cell carcinoma; and SC, squamous cell carcinoma.

**Figure 3:**
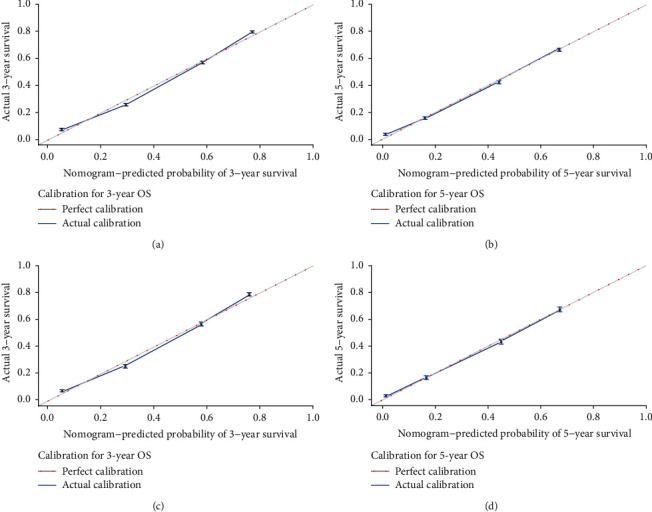
Calibration plots of nomogram in both groups. (a and b). The nomogram was calibrated in the training group by predicting the 3- and 5-year survival. (c and d). The nomogram was also calibrated in the validation group by predicting the 3- and 5-year survival. All results showed better fitting effects.

**Figure 4:**
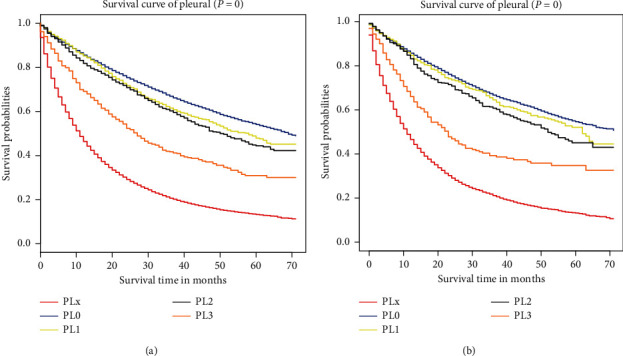
Kaplan–Meier curve analysis in both groups. (a) Risk classification of patient survival with the different degrees of VPI in the training group (log-rank *P* value for trend <0.0001). (b) Risk classification of patient survival with the different degrees of VPI in the validation group (log-rank *P* value for trend <0.0001). Abbreviation: VPI, visceral pleural invasion.

Based on these results, these factors were determined to be independent prognostic factors and then incorporated into the construction of the nomogram predicting 3- and 5-year overall survival (OS) in the training group (Figure 2). This nomogram predictive model revealed that *M* stage had the biggest impact on prognosis, followed by the extent of VPI, histology type, *T* stage, tumor grade, *N* stage, age, gender, pleural, and race. The score of each prognostic factor was identified using the point scale drawn by the intersection of the vertical line from each variable to the point axis. Next, the 3- and 5-year survival probabilities were acquired by adding the score of each prognostic factor. Higher scores among patients correlated with decreased survival. The C-index was 0.772 (95% confidence interval (CI): 0.770–0.774) in the training group and was 0.769 (95% CI: 0.765–0.773) in the validation group. These results ultimately displayed adequate discrimination ability in the prediction of NSCLC patients' absolute risk. The calibration curves, which were validated using the bootstrap resampling method, indicated that the predictive power of the nomogram was in accordance with the actual observed values in both groups (Figure 3). The correlation between the predictive power of OS and the different subdivision levels of VPI shown in Figure 4, which was depicted using Kaplan–Meier curves, could display the survival differences in both the training and the validation groups.

## 4. Discussion

The incidence rate of VPI accounts for approximately 11.5% of NSCLC patients and varies between different histological types [14]. According to the 7th edition of the AJCC/UICC TNM staging system, stage T1a (≤2 cm) and stage T1b (>2 cm and ≤3 cm) can be classified based on the tumor size, whereas VPI confirmed by the pathology finding in NSCLC patients with stage T1 (≤3 cm) can be upgraded to stage T2a. Nonetheless, the detailed subdivision levels of VPI are not incorporated into the *T* stage in the TNM staging system (8th edition). Some [15–17] but not all [18, 19] studies showed that NSCLC patients with VPI had a worse prognosis than those without VPI. Moreover, due to the presence of an independent risk factor for visceral pleural invasion, the relationship between tumor staging and tumor sizes remained controversial. The application of pleural biopsy or medical thoracoscopy is the optional method to estimate the extent of VPI. The clinician often considers various limiting factors such as the increasing risk of malignancy, expertise, surgical complication, and so on. Thus, the extent of VPI can be predicted by imaging examinations, such as B-ultrasound, computed tomography, and other methods. However, the single prediction method and the increase of medical cost may influence diagnostic accuracy and wide clinical practice. Combination with other simple and effective methods has meaningful clinical application value. Therefore, we performed this study to establish a simple predictive tool that can assist clinicians to make the preliminary screening.

Nomograms are prognostic tools that make complex statistical models simpler with terse diagrams, which supply more exact and understandable prognosis predicting results and are widely applied in clinical practice [20, 21]. Predictive models based on large sample data from the SEER database might be less prone to the bias that is selected by the null hypothesis. Meanwhile, due to the lack of details of population-based data, the analyses of the nomogram model derived from the SEER database might be more accurate and more likely to conquer bias through institutional practice [22]. Many clinical research works [23–26] established nomogram prognostic models to provide survival counseling and follow-up strategy-making. These results demonstrated the efficiency of the nomogram predictive tool in clinical practices. Considering the friendly clinician-oriented interface and accurate predictions, therefore, we performed nomograms, which were derived from the SEER database, to predict the correlation with the subdivision level of VPI and the OS of NSCLC patients. We selected the independent prognostic factors using univariate and multivariate Cox proportional hazards regressions to construct the nomogram model. The OS, defined as the time from diagnosis to death of any cause, was assessed by the Kaplan–Meier method and tested by the log-rank test. Consistent with many previous studies [15, 18, 27], the negative correlation between the subdivision level of VPI and the OS of NSCLC patients was revealed. Meanwhile, we found that the subdivision level of VPI could add additional predicting information for OS beyond the other clinical parameters, including age, gender, race, histopathology, and so on, indicating that subdivision of VPI is an independent and strong predictor. In this nomogram prognostic model, a C-index of greater than 0.7 in both the training and the validation groups displayed adequate discrimination ability, and the calibration plots showed good consistency between the predicted and the actual observed values.

Many clinical studies have shown that NSCLC patients with VPI have poorer survival as compared with those without. The possible factors for poor prognosis are associated with higher involvement in mediastinal lymph nodes [28–30]. On the other hand, lung cancer cells under the pleural are more likely to invade the pleural layer rapidly through the flow of pleural effusions in the pleural cavity. Once diaphragmatic lymph nodes have been involved, malignant cells will further be drained to mediastinal lymphatic vessels. In this way, cervical venous circulation is easier to be invaded in the spreading of malignant cells and facilitates metastasis. Malignant tumor cells have increasingly aggressive and progressive biological characteristics with the increasing level of VPI and contribute to adverse outcomes for these NSCLC patients. Some studies have revealed the link between marital status and survival in cancer patients. Meanwhile, several potential mechanisms may explain the correlation. First, patients who are married have less distress and depression than unmarried patients after a diagnosis of cancer, as a partner can share the emotional burden and provide the appropriate social support. Chronic stress, loneliness, and depression can downregulate the immune responses; stimulate tumor angiogenesis; and increase tumor burden and invasiveness [31–34]. Second, patients with emotional and financial support from their spouses or children had better compliance from doctors [35].

There are several limitations in this population-based study. All of the data from the SEER database, which has a retrospective nature, contain bias that should be taken into consideration. First, the different medical agencies and professionals, including pathologists, surgeons, and so on, influence the detection rates and the quality of pleural invasion. Moreover, the SEER database fails to provide other information regarding NSCLC patients with VPI including the method of detection, complications, pulmonary function, and treatment method, which could generate underlying bias as well. Thus, it is useful to improve the accuracy of predictive models by incorporating novel predictors and introducing competing risk models. Moreover, the inclusion of the degree of VPI of early-stage NSCLC patients might also be a good candidate to control the confounding factors. Second, it is a common issue that improved accuracy of the predictive model is usually accompanied by a compromise between the increasing complexity of predictive factors and the decreasing understandability of the model during the modeling process of the nomogram. Considering the aforementioned, variables of clinical importance and high repeatable practicability would be preferred. Moreover, the nomogram itself needs to be confirmed using calibration plots and the C-index due to its uncertainty. Nonetheless, the nomogram is a powerful supplement for clinician judgment and clinical decision-making. Third, occult pleural metastases, which cannot be assessed by routine pathological examination, can only be detected during thoracotomy. Considering this study's retrospective nature, the selection bias could not be avoided. In this study, whether patients diagnosed as PL0 actually have occult pleural metastases is still unclear. Besides this, patients who displayed no signs of pleural invasion based on clinical and/or radiographic judgment cannot exclude the possibility of occult micrometastases. Risk factors of these patients were relatively higher than those diagnosed with VPI. Fourth, based on the SEER database, we randomly divided the data into the training and the validation groups at the ratio of 7:3. This method of nomogram construction and validation is common, whereas further external verification was not available. Hence, we will focus on the data from multiple medical centers in further research to perform the validation of the external cohort.

## 5. Conclusion

We developed and validated a population-based nomogram model to predict survival differences among the different subdivision levels of VPI in NSCLC patients. This study provides a novel perspective that helps clinicians determine survival prognosis and establish personalized treatment strategies for NSCLC patients with varying degrees of VPI, which is an effective supplement to traditional TNM staging.

## Figures and Tables

**Table 1:** Characteristics of NSCLC patients with the different subdivision levels of VPI.

Variables	All patients *N* = (87,045)	Training cohort *N* = (60,933)	Validation cohort *N* = (26,112)
	*N* (%)	*N* (%)	*N* (%)
*Sex*			
Female	42,199 (48.5)	29,550 (48.5)	12,649 (48.4)
Male	44,846 (51.5)	31,383 (51.5)	13,463 (51.6)

*Age*(*years*)			
<60	15,510 (17.8)	10,813 (17.7)	4,697 (18.0)
≥60	71,535 (82.2)	50,120 (82.3)	21,415 (82.0)

*Race*			
Black	9,583 (11.0)	6,758 (11.1)	2,825 (10.8)
White	71,259 (81.9)	49,896 (81.9)	21,363 (81.8)
Others	6,203 (7.1)	4,279 (7.0)	1,924 (7.4)

*Marital*			
Married	46,595 (53.5)	32,584 (53.5)	14,011 (53.7)
Divorced	10,766 (12.4)	7,584 (12.4)	3,182 (12.1)
Single	11,420 (13.1)	7,946 (13.0)	3,474 (13.3)
Widowed	14,727 (16.9)	10,330 (17.0)	4,397 (16.8)
Unknown	3,537 (4.1)	2,489 (4.1)	1,048 (4.0)

*Histology*			
AC	49,530 (56.9)	34,766 (57.1)	14,764 (56.5)
BAC	5,366 (6.1)	3,736 (6.1)	1,630 (6.2)
LCC	1,710 (2.0)	1,202 (2.0)	508 (2.0)
SC	30,439 (35.0)	21,229 (34.8)	9,210 (35.3)

*Grade*			
I	11,539 (13.3)	8,065 (13.2)	3,474 (13.3)
II	35,660 (41.0)	24,918 (40.9)	10,742 (41.1)
III	38,508 (44.2)	26,979 (44.3)	11,529 (44.2)
IV	1,338 (1.5)	971 (1.6)	367 (1.4)

*T stage*			
*T*1	29,605 (34.0)	20,790 (34.1)	8,815 (33.76)
*T*2	25,870 (29.7)	18,047 (29.6)	7,823 (29.96)
*T*3	16,523 (19.0)	11,607 (19.1)	4,916 (18.83)
*T*4	14,972 (17.2)	10,432 (17.1)	4,540 (17.39)

*N stage*			
*N*0	50,120 (57.6)	35,040 (57.5)	15,080 (57.8)
*N*1	8,122 (9.4)	5,736 (9.4)	2,476 (9.5)
*N*2	21,968 (25.2)	15,446 (25.4)	6,522 (25.0)
*N*3	6,745 (7.8)	4,711 (7.7)	2,034 (7.7)

*M stage*			
*M*0	63,034 (72.4)	44,062 (72.3)	18,972 (72.7)
*M*1	24,011 (27.6)	16,871 (27.7)	7,140 (27.3)

*Pleural*			
PLX	45,747 (52.6)	31,976 (52.5)	13,771 (52.7)
*PL*0	34,690 (39.9)	24,389 (40.0)	10,301 (39.4)
*PL*1	2,977 (3.4)	2,064 (3.4)	913 (3.5)
*PL*2	2,529 (2.9)	1,763 (2.9)	766 (3.0)
*PL*3	1,102 (1.3)	741 (1.2)	361 (1.4)

Note: AC: adenocarcinoma; BAC: bronchioalveolar carcinoma; LCC: large-cell carcinoma; SC: squamous cell carcinoma.

**Table 2:** Univariate and multivariate Cox regression analysis of the training group.

Variable	Univariate analysis	Multivariate analysis	
	*P*-value	HR (95% CI)	*P*-value
*Sex*	<0.001		
Female		Reference	
Male		1.370 (1.332–1.410)	<0.001

*Age*(*years*)	<0.001		
<60		Reference	
≥60		1.374 (1.324–1.427)	<0.001

*Race*	<0.001		
Black		Reference	
White		1.039 (0.997–1.084)	0.0708
Other		0.820 (0.767–0.877)	<0.001

*Marital Status*	<0.001		
Married		Reference	
Divorced		1.203 (1.153–1.255)	<0.001
Single		1.172 (1.124–1.223)	<0.001
Widowed		1.371 (1.320–1.424)	<0.001
Unknown		1.084 (1.008–1.165)	0.0287

*Histology*	<0.001		
AC		Reference	
BAC		0.702 (0.649–0.759)	<0.001
LCC		1.388 (1.262–1.528)	<0.001
SC		1.193 (1.158–1.228)	<0.001

*Grade*	<0.001		
I		Reference	
II		1.204 (1.141–1.271)	<0.001
III		1.467 (1.390–1.547)	<0.001
IV		1.453 (1.293–1.634)	<0.001

*T stage*	<0.001		
*T*1		Reference	
*T*2		1.395 (1.339–1.454)	<0.001
*T*3		1.642 (1.571–1.717)	<0.001
*T*4		1.745 (1.667–1.826)	<0.001

*N stage*	<0.001		
** ** *N*0		Reference	
** ** *N*1		1.278 (1.228–1.330)	<0.001
** ** *N*2		1.515 (1.472–1.559)	<0.001
** ** *N*3		1.453 (1.395–1.514)	<0.001

*M stage*	<0.001		
*M*0		Reference	
*M*1		2.427 (2.361–2.494)	<0.001

*Pleural*	<0.001		
PLX		Reference	
*PL*0		0.499 (0.484–0.515)	<0.001
*PL*1		0.465 (0.430–0.503)	<0.001
*PL*2		0.478 (0.441–0.519)	<0.001
*PL*3		0.605 (0.546–0.670)	<0.001

## Data Availability

The data used to support the findings of this study are available from the corresponding author upon request.
